# Association Between Serum Long-Chain n-3 and n-6 Polyunsaturated Fatty Acid Profiles and Glomerular Filtration Rate Assessed by Serum Creatinine and Cystatin C Levels in Japanese Community-Dwellers

**DOI:** 10.2188/jea.JE20140093

**Published:** 2015-04-05

**Authors:** Aya Higashiyama, Yoshimi Kubota, Mikio Marumo, Masami Konishi, Yoshiko Yamashita, Kunihiro Nishimura, Yoshihiro Fukuda, Tomonori Okamura, Ichiro Wakabayashi

**Affiliations:** 1 Department of Environmental and Preventive Medicine, Hyogo College of Medicine, Nishinomiya, Hyogo, Japan; 2 Department of Preventive Medicine and Epidemiologic Informatics, National Cerebral and Cardiovascular Center, Suita, Osaka, Japan; 3 Department of Research and Development of Preemptive and Preventive Medicine, Institute of Biomedical Research and Innovation, Foundation for Biomedical Research and Innovation, Kobe, Japan; 4 Sasayama City Office, Sasayama, Hyogo, Japan; 5 Department of General Medicine and Community Health Science, Hyogo College of Medicine, Sasayama, Hyogo, Japan; 6 Department of Preventive Medicine and Public Health, School of Medicine, Keio University, Tokyo, Japan

**Keywords:** epidemiology, (EPA+DHA):AA, population-based study

## Abstract

**Background:**

Plasma concentration of n-3 polyunsaturated fatty acids (PUFAs) has been reported to be associated with renal function in Western populations. However, few studies have investigated the association between serum long-chain n-3 and n-6 PUFA profiles and renal function in a Japanese population with high marine-derived long-chain n-3 PUFA intake.

**Methods:**

A cross-sectional study was performed in 549 Japanese rural community-dwellers aged 40 to 64 years. In adjusted analysis of covariance, we assessed the relationship between estimated glomerular filtration rate (eGFR) and tertiles of serum long-chain n-3 and n-6 PUFA profiles ([eicosapentaenoic acid {EPA} + docosahexaenoic acid {DHA}]:arachidonic acid [AA]). GFR was estimated by Japanese specific equations using serum creatinine and cystatin C (eGFR_cre_ and eGFR_cys_). Using multivariate-adjusted linear regression models, we also assessed the relationships between eGFRs and several n-3 and n-6 PUFAs, which have been suggested to be associated with renal function.

**Results:**

In all participants, higher dietary fish intake as assessed by a semi-quantitative questionnaire was associated with higher serum value of (EPA+DHA):AA. Participants in the higher (EPA+DHA):AA tertiles had non-significantly higher eGFR_cre_ and significantly higher eGFR_cys_ (*P* = 0.016). In addition, eGFR_cys_ in T_2_+T_3_ of (EPA+DHA):AA was significantly higher than that in T_1_ (adjusted mean eGFR_cys_, T_1_: 87 ml/min/1.73 m^2^, T_2_+T_3_: 91 ml/min/1.73 m^2^; *P* < 0.01). Among the PUFAs, only (EPA+DHA) was significantly associated with eGFR_cys_.

**Conclusions:**

Serum **(**EPA+DHA):AA, which reflects an individual’s fish intake, might be associated with eGFR_cys_ in Japanese community-dwellers.

## INTRODUCTION

N-3 polyunsaturated fatty acids (PUFAs) have been suggested to be protective against the development of renal dysfunction. According to a previous community-based study in Italy, plasma concentration of n-3 PUFAs was inversely associated with age-associated decline in estimated glomerular filtration rate (eGFR).^1^ The Japanese population is unique because it has particularly high fish intake; consequently, Japanese people tend to have high serum long-chain n-3 PUFA levels,^2^ which may be associated with low risk of coronary artery disease.^3^^,^^4^ However, the relationship between serum long-chain n-3 PUFA levels and renal function has not been investigated in Japanese community-dwellers.

Arachidonic acid (AA), which is classified as an n-6 PUFA, has been considered to have inflammatory and thrombotic effects because many (though not all) eicosanoids derived from AA are considered to be inflammatory, whereas EPA- and DHA-derived eicosanoids are considered to be protective against inflammation induced by AA.^5^ Accordingly, previous studies in Japanese patients have investigated the relationship between cardiac events and serum n-3 PUFA:AA ratios, which are markers for balance of n-3 PUFAs and AA.^5^ These studies have shown that higher EPA:AA and (EPA+DHA):AA ratios were associated with lower risk of cardiac events.^5^^–^^7^ However, few community-based epidemiological studies have investigated the relationship between kidney function and long-chain n-3 PUFA:AA ratios.

The Japanese Society of Nephrology has developed 2 equations to estimate GFR, using serum creatinine (Cre) and cystatin C (Cys C) levels.^8^ Serum Cys C is currently being considered as a potential replacement for Cre as a filtration marker because it is not affected by dietary intake and muscle mass.^9^^,^^10^

To investigate the relationships between eGFR and serum long-chain n-3 and n-6 PUFA profiles in community-dwellers, we performed a cross-sectional study in 549 Japanese men and women aged 40–64 years. GFR was estimated by 2 equations for the Japanese population, using serum Cre and Cys C.

## METHODS

### Study participants

The data from the baseline survey of the Sasayama study were analyzed. The Sasayama study is a population-based cohort study in which the endpoints are increased medical expenditures, worsening of quality of life, or cerebral and cardiovascular disease (CVD) risk factors, such as hypertension, diabetes mellitus, and dyslipidemia.

The study participants consisted of Japanese national health insurance (NHI) beneficiaries living in Sasayama City in Western Japan’s Hyogo Prefecture who had undergone a medical examination between May 2012 and February 2013. The NHI system is one of the insurance systems in Japan, which is for non-employees, such as self-employed individuals, farmers, fishermen, and their dependents. During this time period, a total of 1131 NHI beneficiaries aged 40–64 years underwent a medical examination, and 675 individuals agreed to participate in the study. Written informed consent was obtained from each participant. Of these 675 participants, 126 were excluded due to 1 or more of the following reasons: non-fasting visit (*n* = 82), missing data (*n* = 37), or triglyceride level ≥400 mg/dL (*n* = 7). The remaining 549 individuals (237 men and 312 women, mean [standard deviation {SD}] age: 57 [7] years) were included in the present study. The present study was approved by the Hyogo College of Medicine Ethics Committee.

### Data collection and standardization

Height and weight while wearing socks and light clothing were measured, and body mass index (BMI) was calculated as weight (kg) divided by height squared (m^2^). Blood pressure was measured using an automatic sphygmomanometer after a 5-minute rest. Hypertension was defined as systolic blood pressure ≥140 mm Hg and/or diastolic blood pressure ≥90 mm Hg and/or use of medication for hypertension.

The participants were asked to respond to questionnaires about lifestyle-related factors, such as medication, smoking (current smoker or not), alcohol consumption (current drinker or not), and fish intake. The questionnaires included question about the frequency of fish intake per week, and the portion size of fish consumed in his or her typical meal using full-scale photos of 80 g of cooked fish. Then, each participant’s total fish intake per week was calculated by summing the values that were calculated by multiplying the frequency and portion size.

Blood samples after an overnight fast were obtained from all participants. Serum total cholesterol (TC), high-density lipoprotein cholesterol (HDL-C), triglycerides, and glucose levels were measured by enzymatic methods. Low-density lipoprotein cholesterol (LDL-C) was calculated by Friedewald’s formula. Diabetes was defined as fasting blood glucose ≥126 mg/dL and/or HbA1c ≥ 6.5% (measured according to National Glycohemoglobin Standardization Program [NGSP] standards) and/or current use of insulin or oral medication for diabetes.

Fatty acid concentrations were measured using gas chromatography (GC-17A; Shimazu Corp, Kyoto, Japan) in the same commissioned clinical laboratory center (SRL Inc., Tokyo, Japan).^11^ Serum total PUFA concentration was calculated as the sum of n-6 PUFA concentration (linoleic acid [LA, 18:2n-6], γ-linolenic acid [18:3n6], dihomo-γ-linolenic acid [20:3n6], and arachidonic acid [AA, 20:4n6]) and n-3 PUFA concentration ([α-linolenic acid [18:3n3], eicosapentaenoic acid [EPA, 20:5n3], docosapentaenoic acid [22:5n3], and docosahexaenoic acid [DHA, 22:6n3]).^2^ Long-chain n-3 PUFAs were calculated as the sum of EPA, docosapentaenoic acid, and DHA.

Serum Cre was measured using the enzymatic method, and serum Cys C was measured using the colloidal gold technique.^12^ GFR (mL/min/1.73 m^2^) was estimated using the following 2 equations, which were developed by the Japanese Society of Nephrology: equation 1: eGFR_cre_ = 194 × Cre^−1.094^ × age^−0.287^ (× 0.739 if female),^8^ and equation 2: eGFR_cys_ = 104 × Cys C^−1.019^ × 0.996^age^ (× 0.929 if female) − 8.^8^^,^^13^

### Statistical analysis

Sex-specific and sex-combined analyses were performed. To show the characteristics of the study participants classified according to tertiles of the (EPA+DHA):AA ratio, mean (SD) or median were calculated for continuous variables, and the percentage was calculated for dichotomous variables. The crude and age- and sex-adjusted geometric means of fish intake per week were compared among the tertiles of the (EPA+DHA):AA ratio with Bonferroni’s correction for multiple post-hoc comparisons.

To investigate which variables among the long-chain n-3 and n-6 PUFA profiles show large standardized coefficients in relation to eGFR_cre_ and eGFR_cys_, linear regression models were used after adjusting for age, sex, BMI, hypertension, diabetes, HDL-C, LDL-C, medication for dyslipidemia, and current smoking and drinking. In these models, the long-chain n-3 and n-6 PUFA profiles included the serum concentrations of EPA, DHA, EPA+DHA, and long-chain n-3 PUFA, as well as EPA:AA, DHA:AA, (EPA+DHA):AA, and long-chain n-3 PUFA:AA ratios.

Among the tertiles of the (EPA+DHA):AA ratio, eGFR_cre_ and eGFR_cys_ were compared by analysis of covariance (ANCOVA) with Bonferroni’s correction for multiple post-hoc comparisons after adjusting for the following confounders: Model 1 included age, sex, BMI, hypertension, diabetes, HDL-C, LDL-C, medication for dyslipidemia, and current smoking and drinking; Model 2 included variables in Model 1 plus log-transformed C-reactive protein (CRP) measured using a high-sensitivity CRP assay. Because the fish intake of Japanese population was generally higher than that providing the maximal preventive effect for CVD in the previous studies,^14^ eGFR_cre_ and eGFR_cys_ were also compared among the participants in the lowest tertile (T_1_) and those in the other tertiles (T_2_+T_3_) of the (EPA+DHA):AA ratio after adjusting for the same confounders mentioned above.

Because several n-3 and n-6 PUFAs have been suggested to be associated with renal function in previous studies,^1^^,^^15^ multiple linear regression models were used to confirm the contribution of serum PUFA concentration to eGFR_cre_ and eGFR_cys_ after adjusting for age, sex, BMI, hypertension, diabetes, HDL-C, LDL-C, medication for dyslipidemia, and current smoking and drinking. Serum concentrations of EPA+DHA, AA, linoleic acid, and α-linolenic acid were included in Model 1, and serum concentrations of EPA+DHA and n-6 PUFA were included in Model 2.

All *P* values were two-tailed, and the significance level was set at *P* < 0.05. The statistical package SPSS 20.0J for Windows (SPSS, Tokyo, Japan) was used to perform the analyses.

## RESULTS

In all participants, the mean (SD) concentration of serum PUFA was 1457.3 (239.0) µg/mL total PUFA, 286.7 (95.8) µg/mL n-3 PUFA, 260.6 (92.5) µg/mL long-chain n-3 PUFA, and 1170.6 (198.6) µg/mL n-6 PUFA. The mean eGFR_cre_ was 73 (13) mL/min/1.73 m^2^, and the mean eGFR_cys_ was 89 (16) mL/min/1.73 m^2^. Eighty-one individuals had chronic kidney disease (CKD) defined by eGFR_cre_ <60 mL/min/1.73 m^2^, and 12 individuals had CKD defined by eGFR_cys_ <60 mL/min/1.73 m^2^.

Table 1 shows the characteristics of the participants according to serum (EPA+DHA):AA tertile in all participants. Age, BMI, and prevalence of hypertension and diabetes were higher in the higher (EPA+DHA):AA tertile. The percentage of medication for dyslipidemia was lower in the higher (EPA+DHA):AA tertile. eTables 1 and 2 show the sex-specific characteristics of the participants. In men, prevalence of smoking was higher in the lowest tertile compared to other groups (43.6% in T_1_, 25.0% in T_2_, and 29.1% in T_3_). Figure shows the relationships between serum (EPA+DHA):AA tertile and geometric mean of fish intake (g/week). The higher (EPA+DHA):AA tertile was significantly associated with higher fish intake (trend *P* < 0.001, *P* < 0.001 between T_1_ and T_2_, and *P* < 0.05 between T_2_ and T_3_). In sex-specific analysis, the results were similar.

**Figure.**
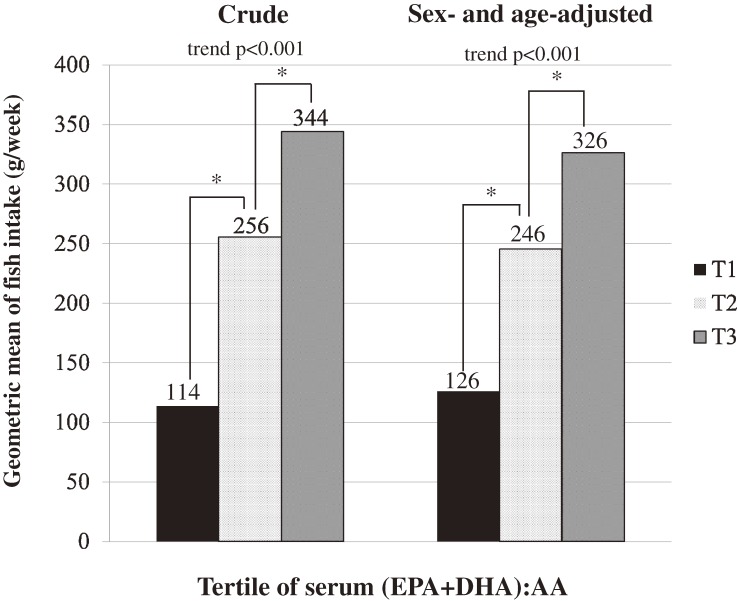
Tertile of serum (EPA+DHA):AA and geometric mean of fish intake. *Bonferroni correction for multiple post-hoc comparisons. Significance between the presented tertiles: *P* < 0.05.

**Table 1.** Characteristics of study participants according to serum (EPA+DHA):AA tertile in the Sasayama study, 2012–2013

	Tertile of (EPA+DHA):AA		

	T_1_ (0.338–0.925)	T_2_ (0.928–1.301)	T_3_ (1.302–3.188)
Number of participants	184	181	184
Sex, % males	44.0	38.1	47.3
Age, years	53 (8)	58 (6)	59 (5)
BMI, kg/m^2^	22.7 (2.9)	23.0 (3.5)	23.2 (3.4)
Systolic blood pressure, mm Hg	121 (16)	128 (19)	130 (19)
Diastolic blood pressure, mm Hg	73 (11)	76 (11)	78 (11)
Hypertension, %	20.7	39.2	39.7
Glucose, mg/dL	95	98	99
Diabetes, %	7.6	7.7	9.8
Total cholesterol, mg/dL	210 (34)	221 (35)	220 (38)
LDL cholesterol, mg/dL	127 (31)	134 (33)	133 (34)
HDL cholesterol, mg/dL	63 (14)	64 (15)	63 (17)
Medication for dyslipidemia, %	19.6	14.9	13.6
Current smoking, %	24.5	13.3	15.2
Current drinking, %	52.2	51.9	55.4
Past or present history of CVD, %	3.3	5.0	3.3
C-reactive protein, mg/L	0.3	0.4	0.4
Fish intake, g/week	114 (5)	256 (2)	344 (2)
Serum N-3 PUFA,^a^ µg/mL	203.3 (48.3)	278.9 (57.9)	377.8 (80.3)
Serum Long chain n-3 PUFA,^b^ µg/mL	180.7 (45.0)	252.0 (56.1)	348.9 (78.8)
Serum EPA, µg/mL	42.2 (15.7)	68.0 (24.9)	111.2 (42.0)
Serum DHA, µg/mL	120.6 (28.8)	162.1 (34.0)	210.5 (42.5)
Serum α-linolenic acid, µg/mL	22.6 (7.8)	26.9 (10.6)	29.0 (10.6)
Serum n-6 PUFA,^c^ µg/mL	1190.3 (190.0)	1181.5 (203.5)	1140.1 (199.6)
Serum AA, µg/mL	224.9 (47.2)	207.7 (45.0)	197.2 (42.1)
Serum linoleic acid, µg/mL	907.1 (163.1)	918.2 (183.0)	894.5 (178.3)
Serum creatinine, mg/dL	0.76 (0.21)	0.74 (0.15)	0.76 (0.17)
eGFR_cre_, mL/min/1.73 m^2^	74 (13)	73 (12)	73 (14)
Serum cystatin C, mg/L	0.85 (0.18)	0.83 (0.12)	0.85 (0.13)
eGFR_cys_, mL/min/1.73 m^2^	91 (18)	89 (15)	88 (15)

AA, arachidonic acid; BMI, body mass index; CVD, cerebral and cardiovascular disease; DHA, docosahexaenoic acid; EPA, eicosapentaenoic acid; HDL, high-density lipoprotein; LDL, low-density lipoprotein; PUFA, polyunsaturated fatty acid.

Values are means (standard deviations), except glucose and high-sensitivity C-reactive protein levels, which are presented as medians.

Fish intake is presented as geometric mean (SD).

^a^Serum n-3 PUFA: sum of α-linolenic acid, EPA, DHA, and docosapentaenoic acid.

^b^Serum n-6 PUFA: sum of linoleic acid, γ-linolenic acid, dihomo-γ-linolenic acid, and AA.

^c^Long-chain n-3 PUFA: sum of EPA, DHA, and docosapentaenoic acid.

eTable 3 shows the relationships between eGFRs and n-3 and n-6 PUFA profiles in multivariate-adjusted linear regression models. All long-chain n-3 PUFA concentrations and ratios of long-chain n-3 PUFA to AA showed significant relationships with eGFR_cys_. These concentrations and ratios did not show significant relationship with eGFR_cre_; however, n-3 PUFA:AA ratios showed higher coefficients for eGFR_cre_ than n-3 PUFA concentrations.

Table 2 shows the association between serum (EPA+DHA):AA tertiles and eGFR_cre_ in ANCOVA after adjusting for the confounders. In men, higher (EPA+DHA):AA tertiles were associated with higher eGFR_cre_ without statistical significance. In women, adjusted means of eGFR_cre_ were the same among (EPA+DHA):AA tertiles. In all participants, higher (EPA+DHA):AA tertiles were associated with higher eGFR_cre_ without statistical significance. Table 3 shows the association between (EPA+DHA):AA tertiles and eGFR_cys_ in ANCOVA after adjusting for the confounders. In men, higher (EPA+DHA):AA tertiles were significantly associated with higher eGFR_cys_. In women, higher (EPA+DHA):AA tertiles were associated with higher eGFR_cys_ without statistical significance. In all participants, higher (EPA+DHA):AA tertiles were significantly associated with higher eGFR_cys_, and Bonferroni’s correction for multiple post-hoc comparisons showed significant differences between T_1_ and T_2_ (*P* < 0.05) and between T_1_ and T_3_ (*P* < 0.05). In addition, as shown in Table 4, eGFR_cre_ was higher in T_2_+T_3_ than in T_1_ without statistical significance, and eGFR_cys_ was significantly higher in T_2_+T_3_ than in T_1_ in all participants.

**Table 2.** Multivariate-adjusted eGFR_cre_ according to tertile of serum (EPA+DHA):AA ratio in the Sasayama study, 2012–2013

	Tertile of serum (EPA+DHA):AA			*P* value^a^

	T_1_	T_2_	T_3_	
Men				
Number of participants	78	80	79	
Range of (EPA+DHA):AA ratio	0.409–0.904	0.908–1.318	1.323–3.188	
Sex- and age-adjusted mean eGFR_cre_ (95% CI)	72.1 (68.8–75.4)	72.2 (69.1–75.4)	75.2 (72.0–78.5)	0.322
Multivariate-adjusted mean eGFR_cre_ (95% CI) (Model 1)^b^	71.3 (68.0–74.6)	72.5 (69.4–75.6)	75.8 (72.6–79.0)	0.139
Multivariate-adjusted mean eGFR_cre_ (95% CI) (Model 2)^c^	71.3 (68.0–74.6)	72.5 (69.4–75.6)	75.8 (72.6–79.0)	0.147
Women				
Number of participants	103	104	105	
Range of (EPA+DHA):AA ratio	0.338–0.925	0.929–1.282	1.283–2.777	
Sex- and age-adjusted mean eGFR_cre_ (95% CI)	73.2 (70.7–75.7)	73.2 (70.9–75.5)	73.0 (70.7–75.3)	0.989
Multivariate-adjusted mean eGFR_cre_ (95% CI) (Model 1)^b^	73.2 (70.6–75.7)	73.2 (70.9–75.5)	73.1 (70.7–75.4)	0.998
Multivariate-adjusted mean eGFR_cre_ (95% CI) (Model 2)^c^	73.2 (70.6–75.7)	73.1 (70.8–75.5)	73.1 (70.8–75.5)	1.000
Men and women combined				
Number of participants	184	181	184	
Range of (EPA+DHA):AA ratio	0.338–0.925	0.928–1.301	1.301–3.188	
Sex- and age-adjusted mean eGFR_cre_ (95% CI)	72.7 (70.7–74.7)	73.1 (71.2–75.0)	73.7 (71.8–75.6)	0.776
Multivariate-adjusted mean eGFR_cre_ (95% CI) (Model 1)^b^	72.3 (70.3–74.2)	73.3 (71.4–75.2)	74.0 (72.1–75.9)	0.507
Multivariate-adjusted mean eGFR_cre_ (95% CI) (Model 2)^c^	72.2 (70.2–74.2)	73.3 (71.4–75.1)	74.0 (72.1–75.9)	0.476

AA, arachidonic acid; DHA, docosahexaenoic acid; EPA, eicosapentaenoic acid; GFR, glomerular filtration rate.

^a^*P* value for ANCOVA.

^b^Multivariate-adjusted (Model 1): eGFR adjusted for age, sex, BMI, hypertension, diabetes, HDL-C, LDL-C, medication for dyslipidemia, current smoking, and current drinking.

^c^Multivariate-adjusted (Model 2): eGFR adjusted for variables in model 1 plus log-transformed high-sensitivity C-reactive protein.

**Table 3.** Multivariate-adjusted eGFR_cys_ according to tertile of serum (EPA+DHA):AA ratio in the Sasayama study, 2012–2013

	Tertile of serum (EPA+DHA):AA			*P* value^a^

	T_1_	T_2_	T_3_	
Men				
Number of participants	78	80	79	
Range of (EPA+DHA):AA ratio	0.409–0.904	0.908–1.318	1.323–3.188	
Sex- and age-adjusted mean eGFR_cys_ (95% CI)	82.9 (79.5–86.3)	89.5 (86.2–92.7)	90.7 (87.4–94.1)	0.003
Multivariate-adjusted mean eGFR_cys_ (95% CI) (Model 1)^b^	83.9 (80.5–87.2)	88.2 (85.0–91.3)	91.0 (87.8–94.3)	0.015
Multivariate-adjusted mean eGFR_cys_ (95% CI) (Model 2)^c^	84.0 (80.6–87.3)	88.2 (85.1–91.4)	90.9 (87.7–94.1)	0.017
Women				
Number of participants	103	104	105	
Range of (EPA+DHA):AA ratio	0.338–0.925	0.929–1.282	1.283–2.777	
Sex- and age-adjusted mean eGFR_cys_ (95% CI)	89.6 (86.8–92.3)	91.2 (88.7–93.8)	91.2 (88.6–93.8)	0.654
Multivariate-adjusted mean eGFR_cys_ (95% CI) (Model 1)^b^	89.3 (86.6–91.9)	91.5 (89.1–93.9)	91.2 (88.8–93.7)	0.466
Multivariate-adjusted mean eGFR_cys_ (95% CI) (Model 2)^c^	89.2 (86.6–91.8)	91.4 (89.0–93.8)	91.4 (89.0–93.7)	0.454
Men and women combined				
Number of participants	184	181	184	
Range of (EPA+DHA):AA ratio	0.338–0.925	0.928–1.301	1.301–3.188	
Sex- and age-adjusted mean eGFR_cys_ (95% CI)	86.9 (84.7–89.0)	90.7 (88.6–92.8)	90.7 (88.5–92.7)	0.023
Multivariate-adjusted mean eGFR_cys_ (95% CI) (Model 1)^b^	86.9 (84.9–89.0)	90.6 (88.7–92.6)	90.6 (88.7–92.6)	0.021
Multivariate-adjusted mean eGFR_cys_ (95% CI) (Model 2)^c^	86.9 (84.9–88.9)	90.5 (88.6–92.4)	90.8 (88.9–92.7)	0.016

AA, arachidonic acid; DHA, docosahexaenoic acid; EPA, eicosapentaenoic acid; GFR, glomerular filtration rate.

^a^*P* value for ANCOVA.

^b^Multivariate-adjusted (Model 1): eGFR adjusted for age, sex, BMI, hypertension, diabetes, HDL-C, LDL-C, medication for dyslipidemia, current smoking, and current drinking.

^c^Multivariate-adjusted (Model 2): eGFR adjusted for variables in model 1 plus log-transformed high-sensitivity C-reactive protein.

**Table 4.** Multivariate-adjusted eGFR in T_1_ and T_2_+T_3_ of serum (EPA+DHA):AA ratio in the Sasayama study, 2012–2013

	Tertile of serum (EPA+DHA):AA		*P* value^a^

	T_1_ (0.338–0.925)	T_2_+T_3_ (0.928–3.188)	
Number of participants	184	365	
Mean eGFR_cre_ (ml/min/1.73 m^2^)			
Sex- and age-adjusted	72.7 (70.8–74.7)	73.4 (72.0–74.8)	0.598
Multivariate-adjusted (Model 1)^b^	72.3 (70.3–74.3)	73.6 (72.3–75.0)	0.291
Multivariate-adjusted (Model 2)^c^	72.3 (70.3–74.2)	73.6 (72.3–75.0)	0.282
Mean eGFR_cys_ (ml/min/1.73 m^2^)			
Sex- and age-adjusted	86.9 (84.7–89.0)	90.7 (89.2–92.1)	0.006
Multivariate-adjusted (Model 1)^b^	86.9 (84.9–89.0)	90.6 (89.2–92.0)	0.005
Multivariate-adjusted (Model 2)^c^	86.9 (84.9–88.9)	90.6 (89.3–92.0)	0.004

AA, arachidonic acid; DHA, docosahexaenoic acid; EPA, eicosapentaenoic acid; GFR, glomerular filtration rate.

^a^*P* value for ANCOVA.

^b^Multivariate-adjusted (Model 1): eGFR adjusted for age, sex, BMI, hypertension, diabetes, HDL-C, LDL-C, medication for dyslipidemia, current smoking, and current drinking.

^c^Multivariate-adjusted (Model 2): eGFR adjusted for variables in model 1 plus log-transformed high-sensitivity C-reactive protein.

Multivariate-adjusted (Model 2): eGFR adjusted for variables in model 1 plus log-transformed high-sensitivity C-reactive protein.

In addition, eGFRs were also compared among tertiles of fish intake (T_1_, T_2_, and T_3_) in all participants by ANCOVA after adjusting for the confounders described as Model 1. Mean eGFR_cre_ was 72 mL/min/1.73 m^2^ in T_1_, 75 mL/min/1.73 m^2^ in T_2_, and 73 mL/min/1.73 m^2^ in T_3_ (*P* for ANCOVA = 0.204), and mean eGFR_cys_ was 88 mL/min/1.73 m^2^ in T_1_, 90 mL/min/1.73 m^2^ in T_2_, and 90 mL/min/1.73 m^2^ in T_3_ (*P* for ANCOVA = 0.163) (data not shown).

Table 5 shows the standardized coefficients of EPA+DHA and other PUFA concentrations in relation to eGFR_cre_ and eGFR_cys_ in multivariate-adjusted linear regression analysis in all participants. None of the presented PUFA concentrations, including EPA+DHA, were significantly associated with eGFR_cre_; however, EPA+DHA concentration was significantly associated with eGFR_cys_.

**Table 5.** Multivariate-adjusted linear regression models^a^ between eGFR and serum PUFAs, including EPA+DHA, in the Sasayama study, 2012–2013

	Independent variables			

	eGFR_cre_		eGFR_cys_	
	
	Standardized coefficients	*P* value	Standardized coefficients	*P* value
Model 1				
Serum EPA+DHA	−0.012	0.815	0.097	0.025
Serum α-linolenic acid	0.088	0.191	0.015	0.797
Serum AA	0.008	0.862	−0.025	0.541
Serum linoleic acid	−0.144	0.064	−0.016	0.803
Model 2				
Serum EPA+DHA	0.014	0.770	0.096	0.015
Serum n-6 PUFA^b^	−0.055	0.313	−0.022	0.622

AA, arachidonic acid; DHA, docosahexaenoic acid; EPA, eicosapentaenoic acid; GFR, glomerular filtration rate; PUFA, polyunsaturated fatty acids.

^a^Relationships between presented fatty acids and eGFR were evaluated by linear regression model after adjusting for age, sex, BMI, presence of hypertension and diabetes mellitus, serum HDL- and LDL- cholesterol level, medication for dyslipidemia, current smoking and drinking, and log-transformed C-reactive protein.

^b^Serum n-6 PUFA: sum of linoleic acid, γ-linolenic acid, dihomo-γ-linolenic acid, and arachidonic acid.

## DISCUSSION

In the present study, the higher serum (EPA+DHA):AA tertile was significantly associated with higher fish intake in Japanese community-dwelling men and women. Furthermore, especially in men, higher serum (EPA+DHA):AA was significantly associated with higher eGFR_cys_. In all participants, eGFR_cre_ non-significantly increased according to an increase of (EPA+DHA):AA.

To our knowledge, the present study is the first to investigate the relationships between serum long-chain n-3 and n-6 PUFA profile and eGFR_cys_. Because the ratios of n-3 PUFA to AA are considered to be markers for balance of anti-inflammatory and proinflammatory action by n-3 PUFAs and AA,^5^ previous studies among Japanese patients have investigated the relationships between cardiac events and the ratio of n-3 PUFAs to AA. Among patients undergoing coronary angioplasty or hemodialysis, lower EPA:AA ratios were associated with higher risk of acute coronary syndrome,^16^ and lower (EPA+DHA):AA ratios were associated with higher incidence of cardiovascular disease.^5^ However, the relationships between serum long-chain n-3 and n-6 PUFA profiles and GFR estimated by Japanese-specific equations have not been investigated in community-dwellers.

In Western populations, only a few previous studies have investigated the relationships between PUFAs and renal function in community-dwellers. Gopinath et al showed that dietary intake of long-chain n-3 PUFA was inversely associated with the prevalence of CKD in a cross-sectional study of 2600 community-dwellers in Australia.^15^ Lauretani et al showed that participants with higher plasma n-3 PUFA concentration had a significantly lower risk of developing CKD and mortality in a cohort study of 931 community-dwellers.^1^ The results of these previous studies are consistent with those of the present study.

On the other hand, there has been a series of conflicting reports regarding the benefit of fish oil preparations containing n-3 PUFA given to patients with a variety of disease. Hsu et al showed that frequent intake of fish and vegetables correlated significantly with decreased creatinine and marginally with increased GFR estimated by Cre in a cohort study of patients with type 2 diabetes in Taiwan.^17^ According to a meta-analysis of clinical trials by Miller III et al, the decline of GFR was slower in participants with n-3 PUFA supplementation than in control participants, but this effect was not significant, and they concluded that n-3 PUFA supplementation did not ameliorate the decline in GFR. However, they also noted that differences in methods of assessing GFR, such as GFR measured or estimated by serum Cre or 24-h urine Cre clearance, limited the ability to draw conclusions.^18^ Furthermore, serum Cre level is affected by various factors, such as muscle mass and diet.^10^

In the present study, the difference between eGFR_cys_ and eGFR_cre_ in relation to EPA+DHA:AA was especially apparent in men. Thus, muscle mass could be an important factor influencing the relationship between serum EPA+DHA:AA and eGFR_cre_, and eGFR_cys_ might be more useful than eGFR_cre_ when investigating the relationship between eGFR and PUFA profiles. In addition, in the previous studies,^1^^,^^15^^,^^17^^,^^18^ ratios of long-chain n-3 and n-6 PUFAs, such as (EPA+DHA):AA, were not investigated in relation to eGFR. As shown in eTable 3, all long-chain n-3 PUFA concentrations and ratios of long-chain n-3 PUFAs to AA showed significant associations with eGFR_cys_. And although both the concentrations and the ratios did not show significant relationship with eGFR_cre_, the ratio of long-chain n-3 PUFAs to AA showed higher coefficients with eGFR_cre_ than n-3 PUFA concentrations. Accordingly, the present study suggests that ratios of serum long-chain n-3 PUFAs to AA could be useful when investigating the relationship between PUFA profiles and renal function.

The mechanisms by which higher (EPA+DHA):AA ratios protect renal function are still not clear. However, dietary fish oil supplementation has been shown to slow renal disease progression in patients with IgA nephropathy^19^ and to suppress mesangial cell activation and proliferation in animal models.^20^ Therefore, long-chain n-3 PUFAs are considered to attenuate inflammation through several pathways, including those involved in reduction of nitric oxide, downregulation of tumor necrosis factor-α, and modulation of protein kinases.^21^^–^^23^ Furthermore, Minuz et al demonstrated that alterations in cytochrome P450 (CYP)-dependent AA metabolism are associated with the development of vascular and tubular abnormalities in patients with renovascular disease.^24^ Arnold et al showed that EPA and DHA are efficient alternative substrates of AA-metabolizing CYP enzymes, and that dietary EPA/DHA supplementation causes a profound shift in the CYP-eicosanoid profile.^25^ In addition, Cicero et al showed that long-term n-3 PUFA supplementation was associated with significant reduction in blood pressure.^26^

The hypocholesterolemic effects of fish intake have also been reported.^27^ The association between (EPA+DHA):AA ratio and eGFR was independent of the presence of hypertension and cholesterol level; however, these mechanisms could be also associated with the results in the present study. Furthermore, the difference in eGFR between T_1_ and T_3_ was higher in men than in women. According to sex-specific characteristics of the participants (eTable 1), prevalence of smoking in men was high in T_1_. Therefore, unfavorable lifestyles, such as smoking and low fish intake, might be one possible reason for poorer renal function.

Gopinath et al also showed that linolenic acid (18:3n-3; α-linolenic acid) intake was positively associated with the odds of CKD.^15^ In contrast, Lauretani et al showed that a higher plasma concentration of α-linolenic acid, n-6 PUFAs, linoleic acid, and AA were associated with lower decline in Cre clearance.^1^ In the present study, serum concentration of α-linolenic acid, n-6 PUFAs, linoleic acid, and AA did not show significant relationships with eGFR. A previous study reported that serum concentrations of EPA and DHA were generally higher in the Japanese population than in Western populations.^2^ Therefore, in the present study, the relatively higher concentration of EPA+DHA might mask the relationship between renal function and other PUFAs, such as α-linolenic acid, n-6 PUFAs, linoleic acid, and AA.

The significant relationships between the tertile of (EPA+DHA):AA and fish intake in the present study suggest that higher fish intake could be related to higher eGFR. Indeed, the participants with higher fish intake tended to have higher eGFR_cys_ in the present study. According to previous studies, fish consumption differs by country (mean daily fish intake: 71–125 g in Japan, 32 g among Caucasians in the United States [U.S.], and 85–110 g in Norway).^2^^,^^28^^,^^29^ Hallen et al performed an international comparison of CKD prevalence between the participants in the third National Health and Nutrition Examination Survey (NHANES III) in the U.S. and those of a survey (HUNT II) in Norway.^30^ After age standardization, the prevalence of CKD in HUNT II was 9.3%, and the prevalence in NHANES III was 11.0%. Although they did not consider fish consumption in the analyses, the difference in CKD prevalence between the two populations might be partly due to the difference in fish consumption, as well as due to the lower prevalence of diabetes and obesity in Norway. However, the prevalence of CKD is not low in the general Japanese population (12.9%), despite high fish consumption.^8^ This discrepancy is most likely because average life expectancy has been extended^31^; blood pressure is relatively high^4^; and the prevalence of diabetes, hypercholesterolemia, and obesity has increased in the Japanese population.^32^

The present study had several limitations. First, because the study was cross-sectional, causality could not be determined. Second, information about corticosteroid use was not available. Third, sex-specific analyses were difficult due to the small number of participants. Fourth, although Cre clearance and insulin clearance are better markers of renal function, these data were not available in the present study. Finally, body muscle mass might be related to the results of the present study; however, these data were also not available.

In conclusion, serum long-chain n-3 and n-6 PUFA profiles, especially the (EPA+DHA):AA ratio, were significantly associated with GFR estimated by an equation using serum Cys C in Japanese community-dwellers. The results of the present study suggest that increased fish intake or supplementation with long-chain n-3 PUFAs might prevent renal dysfunction in the general population.

## ONLINE ONLY MATERIALS

eTable 1. Characteristics of study participants according to serum (EPA+DHA):AA tertile in men: the Sasayama study, 2012–2013.

eTable 2. Characteristics of study participants according to serum (EPA+DHA):AA tertile in women: the Sasayama study, 2012–2013.

eTable 3. Multivariate-adjusted linear regression models between serum n-3 and n-6 PUFA profiles and estimated GFRs by 2 different equations.
