# Supplementary material for: Association Between Serum Long-Chain n-3 and n-6 Polyunsaturated Fatty Acid Profiles and Glomerular Filtration Rate Assessed by Serum Creatinine and Cystatin C Levels in Japanese Community-Dwellers
Source: J Epidemiol. 2015 Apr 5;25(4):303–11. doi: 10.2188/jea.JE20140093 (PMC4375285; doi:10.2188/jea.JE20140093)
Supplement: eTable 1. [file je-25-303-s001.pdf]

eTable 1. Characteristics of study participants according to serum (EPA+DHA):AA tertile in men in the Sasayama study, 2012-2013

	Tertile of (EPA+DHA):AA		
	T_1 (0.409-0.904)	T_2 (0.908-1.318)	T_3 (1.323-3.188)
Number of participants	78	80	79
Age, years	53 (7)	57 (7)	58 (6)
BMI, kg/m^2	23.3 (3.1)	23.5 (2.9)	23.7 (3.2)
Systolic blood pressure, mm Hg	124 (16)	129 (16)	131 (19)
Diastolic blood pressure, mm Hg	77 (11)	80 (10)	80 (11)
Hypertension, %	29.5	38.8	43.0
Glucose, mg/dL	100	101	100
Diabetes, %	11.5	12.5	16.5
Total cholesterol, mg/dL	204 (35)	215 (32)	205 (33)
LDL cholesterol, mg/dL	124 (31)	125 (30)	122 (32)
HDL cholesterol, mg/dL	57 (13)	62 (17)	57 (14)
Medication for dyslipidemia, %	17.9	6.3	5.1
Current smoking, %	43.6	25.0	29.1
Current drinking, %	67.9	86.3	75.9
History of CVD, %	3.8	6.3	6.3
High-sensitivity C-reactive protein, mg/L	0.5	0.4	0.4
Serum n-3 PUFA,[a] μg/mL	201.9 (48.1)	284.5 (69.6)	375.7 (85.0)
Serum n-6 PUFA,[b] μg/mL	1179.9 (208.9)	1180.2 (232.9)	1077.4 (192.2)
Serum long chain n-3 PUFA,[c] μg/mL	178.7 (44.8)	256.4 (67.3)	347.8 (82.1)
Fish intake, g/week	97 (5)	234 (3)	340 (2)
Serum creatinine, mg/dL	0.90 (0.24)	0.88 (0.13)	0.86 (0.16)
$eGFR_{cre}$, mL/min/1.73 m^2	73 (15)	72 (13)	74 (16)
Serum cystatin C, mg/L	0.94 (0.21)	0.87 (0.12)	0.88 (0.14)
$eGFR_{cys}$, mL/min/1.73 m^2	85 (17)	89 (15)	89 (16)

AA, arachidonic acid; BMI, body mass index; CVD, cerebral and cardiovascular disease; DHA, docosahexaenoic acid; EPA, eicosapentaenoic acid; HDL, high-density lipoprotein; LDL, low-density lipoprotein; PUFA, polyunsaturated fatty acid.

Values are means (standard deviations) unless specified otherwise. Glucose and high-sensitivity C-reactive protein levels are presented as medians. Fish intake is presented as geometric mean (SD).

[a] N-3 PUFA: the sum of linolenic acid, EPA, DHA, and docosapentaenoic acid.

[b] N-6 PUFA: the sum of linoleic acid, γ-linolenic acid, dihomo-γ-linolenic acid, and AA.

[c] Long-chain n-3 PUFA: the sum of EPA, DHA, and docosapentaenoic acid.
