# Supplementary material for: Association Between Serum Long-Chain n-3 and n-6 Polyunsaturated Fatty Acid Profiles and Glomerular Filtration Rate Assessed by Serum Creatinine and Cystatin C Levels in Japanese Community-Dwellers
Source: J Epidemiol. 2015 Apr 5;25(4):303–11. doi: 10.2188/jea.JE20140093 (PMC4375285; doi:10.2188/jea.JE20140093)
Supplement: eTable 2. [file je-25-303-s002.pdf]

eTable 2. Characteristics of study participants according to serum (EPA+DHA):AA tertile in women in the Sasayama study, 2012-2013

	Tertile of (EPA+DHA):AA		
	T_1 (0.338-0.925)	T_2 (0.929-1.282)	T_3 (1.283-2.777)
Number of participants	103	104	105
Age, years	52 (8)	59 (5)	59 (5)
BMI, kg/m^2	22.2 (2.8)	22.6 (3.7)	22.8 (3.7)
Systolic blood pressure, mm Hg	119 (15)	128 (21)	130 (18)
Diastolic blood pressure, mm Hg	70 (10)	75 (12)	76 (11)
Hypertension, %	13.6	38.5	38.1
Glucose, mg/dL	93	98	96
Diabetes, %	4.9	4.8	3.8
Total cholesterol, mg/dL	214 (33)	226 (36)	231 (38)
LDL cholesterol, mg/dL	129 (32)	139 (34)	142 (34)
HDL cholesterol, mg/dL	67 (13)	67 (14)	67 (17)
Medication for dyslipidemia, %	20.4	20.2	21.9
Current smoking, %	10.7	4.8	3.8
Current drinking, %	40.8	29.8	35.2
History of CVD, %	2.9	3.8	1.0
High-sensitivity C-reactive protein, mg/L	0.2	0.4	0.4
Serum n-3 PUFA,[a] μg/mL	201.8 (46.2)	280.5 (53.5)	373.9 (79.8)
Serum n-6 PUFA,[b] μg/mL	1192.5 (168.1)	1189.3 (188.3)	1186.4 (189.6)
Serum long chain n-3 PUFA,[c] μg/mL	179.9 (42.9)	254.2 (51.6)	344.5 (79.2)
Fish intake, g/week	129 (5)	279 (2)	334 (2)
Serum creatinine, mg/dL	0.66 (0.09)	0.66 (0.09)	0.66 (0.10)
$eGFR_{cre}$, mL/min/1.73 m^2	75 (12)	72 (12)	72 (12)
Serum cystatin C, mg/L	0.78 (0.12)	0.81 (0.10)	0.81 (0.12)
$eGFR_{cys}$, mL/min/1.73 m^2	96 (18)	89 (13)	88 (15)

AA, arachidonic acid; BMI, body mass index; CVD, cerebral and cardiovascular disease; DHA, docosahexaenoic acid; EPA, eicosapentaenoic acid; HDL, high-density lipoprotein; LDL, low-density lipoprotein; PUFA, polyunsaturated fatty acid.

Values are means (standard deviations) unless specified otherwise. Glucose and high-sensitivity C-reactive protein levels are presented as medians. Fish intake is presented as geometric mean (SD).

[a] N-3 PUFA: the sum of linolenic acid, EPA, DHA, and docosapentaenoic acid.

[b] N-6 PUFA: the sum of linoleic acid, γ-linolenic acid, dihomo-γ-linolenic acid, and AA.

[c] Long-chain n-3 PUFA: the sum of EPA, DHA, and docosapentaenoic acid.
