# Supplementary material for: Association Between Serum Long-Chain n-3 and n-6 Polyunsaturated Fatty Acid Profiles and Glomerular Filtration Rate Assessed by Serum Creatinine and Cystatin C Levels in Japanese Community-Dwellers
Source: J Epidemiol. 2015 Apr 5;25(4):303–11. doi: 10.2188/jea.JE20140093 (PMC4375285; doi:10.2188/jea.JE20140093)
Supplement: eTable 3. [file je-25-303-s003.pdf]

eTable 3. Multivariate-adjusted linear regression models[a] between serum n-3 and n-6 PUFA profiles and estimated GFRs by 2 different equations

	**Independent variables**			
	**$eGFR_{cre}$**		**$eGFR_{cys}$**	
	**standardized coefficient**	**P value**	**standardized coefficient**	**P value**
**Dependent variables**				
Serum EPA	0.010	0.826	0.082	0.037
Serum DHA	0.011	0.817	0.091	0.022
Serum EPA+DHA	0.011	0.808	0.094	0.018
Serum long-chain n-3 PUFA[b]	0.015	0.752	0.093	0.020
Serum EPA/AA	0.016	0.719	0.078	0.039
Serum DHA/AA	0.037	0.417	0.087	0.024
Serum EPA+DHA/AA	0.030	0.510	0.090	0.020
Serum long-chain n-3 PUFA[b]:AA	0.034	0.454	0.089	0.021

AA, arachidonic acid; DHA, docosahexaenoic acid; EPA, eicosapentaenoic acid; GFR, glomerular filtration rate; PUFA, polyunsaturated fatty acids.

[a] Relationships between each fatty acid profile and eGFR were evaluated separately by linear regression model after adjusting for age, sex, BMI, hypertension, diabetes mellitus, serum HDL- and LDL-cholesterol level, medication for dyslipidemia, and current smoking and drinking.

[b] Long-chain n-3 PUFA: sum of EPA, DHA, and docosapentaenoic acid.
